# Iodine-induced synthetic method and pharmacokinetic study of cis- and trans-crocetin

**DOI:** 10.3389/fphar.2024.1364286

**Published:** 2024-04-09

**Authors:** Boshen Li, Yuxuan Zhang, Zhiqiang Yang, Xiaolin Li, Jun Yang, Kai Luo, Renjie Wang, Chengrong Xiao, Maoxing Li, Yue Gao

**Affiliations:** ^1^ Department of Pharmaceutical Sciences, Beijing Institute of Radiation Medicine, Beijing, China; ^2^ College of Pharmacy, Gansu University of Chinese Medicine, Lanzhou, China; ^3^ Institutes of Chemical Technology, Northwest Minzu University, Lanzhou, China; ^4^ National Key Laboratory of Kidney Diseases, Beijing, China

**Keywords:** crocetin, iodine-induced, pharmacokinetic, cis isomers, cis-crocetin

## Abstract

**Objective:**

This experiment aimed to obtain the relatively rare cis-crocetin isomer from natural plants, which predominantly exist in the more stable all-trans configuration. This was achieved through iodine-induced isomerization, followed by purification and structural identification. The study also aimed to compare the pharmacokinetic differences between cis- and trans-crocetin *in vivo*.

**Methods:**

Trans-crocetin of high purity was extracted by hydrolysis from gardenia yellow pigment. Cis-crocetin was then synthesized through an optimized electrophilic addition reaction induced by elemental iodine, and subsequently separated and purified *via* silica gel column chromatography. Structural identification of cis-crocetin was determined using IR, UV, and NMR techniques. *In vivo* pharmacokinetic studies were conducted for both cis- and trans-crocetin. In addition to this, we have conducted a comparative study on the *in vivo* anti-hypoxic activity of trans- and cis-crocetin.

**Results:**

Under the selected reaction conditions using DMF as the solvent, with a concentration of 2.5 mg/mL for both trans-crocetin and the iodine solution, and adjusting the illumination time according to the amount of trans-crocetin, the rate of iodine-induced isomerization was the fastest. Cis-crocetin was successfully obtained and, after purification, its structure was identified and found to be consistent with reported data. Cis-crocetin exhibited a faster absorption rate and higher bioavailability, and despite its shorter half-life, it could partially convert to trans-crocetin in the body, thereby extending the duration of the drug’s action within the body to some extent.

**Conclusion:**

This study accomplished the successful preparation and structural identification of cis-crocetin. Additionally, through pharmacokinetic studies, it uncovered notable variations in bioavailability between cis- and trans-crocetin. These findings serve as a solid scientific foundation for future functional research and practical applications in this field.

## 1 Introduction

**FIGURE 1:**
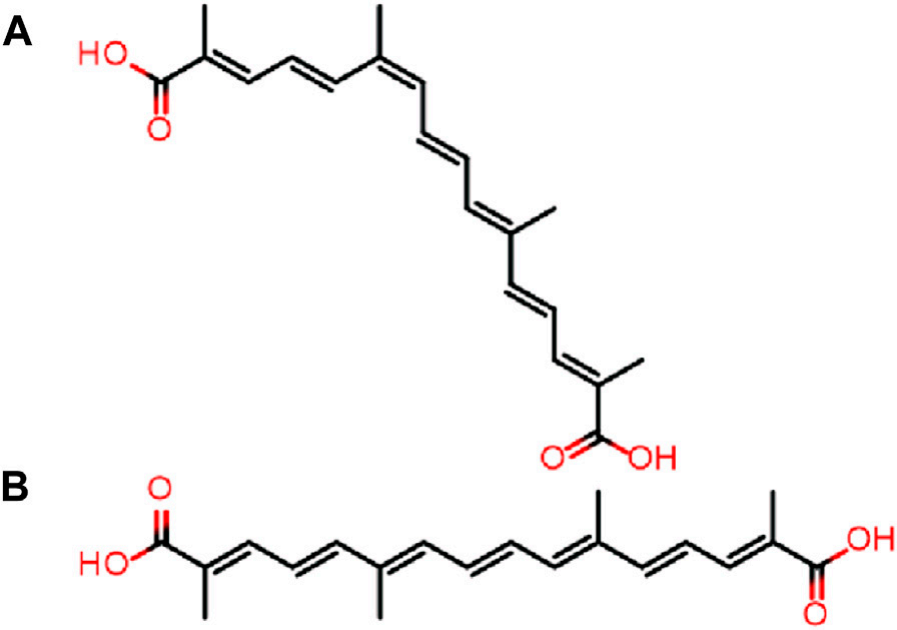
Structure of cis-crocetin **(A)** and trans-crocetin **(B)**.

Crocetin, a biologically active substance, is typically extracted from saffron, a plant species of the iris family scientifically known as *Crocus sativus* L. It can also be obtained from the fruit of *Gardenia jasminoides* Ellis, a member of the gardenia family. Crocetin is a low molecular weight carotenoid compound characterized by a diterpene symmetric structure with seven double bonds and four side chain methyl groups, which classify it as a carotenoid. Its polyunsaturated conjugated enoic acid structure features a carboxyl group attached to each terminal side, as illustrated in Figure 1. Numerous studies have indicated that crocetin possesses significant potential in areas such as anti-tumor, anti-cardiovascular disease, liver and kidney protection, anti-inflammation, retina protection, antioxidant, and myocardial protection (Guo et al., 2021).

Previous research has revealed that gardenia contains both cis and trans conformations of crocetin. However, the majority of studies reported in the literature regarding the preparation process and pharmacological activity of crocetin are mainly focused on trans-crocetin. In contrast, studies on cis-crocetin are scarce. Furthermore, the content of trans isomers of crocetin in gardenia is significantly higher than that of cis. This may be attributed to the significant spatial resistance produced by the large groups near the double bond when in the cis structure. This resistance can lead to instability in the cis structure, resulting in the natural occurrence of crocetin in a more stable all-trans configuration, with lower levels of the cis isomer generally observed in plants (Andersson et al., 2001; Honda, 2022).

It has been observed that the presence of cis–trans isomerism can result in different physicochemical properties and pharmacological effects. For instance, the cis form may exhibit improved bioavailability and potency (Melendez-Martinez, 2022). Boileau et al. (2002) conducted a study on lycopene and found that the cis-labile isomer was more abundant than the trans-labile isomer in both humans and F344 rats. Moreover, the bioavailability of the cis form was significantly higher than that of the trans form. The research workers proposed that the enhanced absorption of cis isomers could be attributed to their shorter molecular lengths, higher solubility in mixed micelles, and reduced tendency to aggregate.

Furthermore, there have been reports in the literature suggesting that crocetin may undergo conformational changes within living organisms. Chryssanthi et al. (2011) investigated the *in vivo* pharmacokinetics of crocetin, which is the active component in saffron tea, by administering the tea to healthy individuals. They discovered that crocetin underwent isomerization in the human body, with cis-crocetin being detected in 75% of the individuals. The cis configuration accounted for 25%–50% of the total crocetin content. Additionally, during their research on the anti-hypoxic activity of trans-crocetin, the group observed the appearance of varying amounts of cis-crocetin in different tissues of rats after oral administration of trans-crocetin. Consequently, further investigations are necessary to determine the potential pharmacological effects of cis-crocetin *in vivo*. Subsequent experiments should also examine and compare the physicochemical properties, bioavailability, and efficacy of cis-crocetin in relation to trans-crocetin.

In this study, we present a method for obtaining high-purity cis-crocetin from gardenia yellow pigment, using iodine induction to induce the structural transformation of trans-crocetin into its cis form. Subsequently, the isolated cis-crocetin was purified through silica gel column chromatography. To determine the optimal conditions for the entire process, we assessed the influence of the reaction solvent, drug concentration, and iodine concentration on the conversion, conversion rate, and isomerization rate, respectively, during the iodine-induced process. The screening of these optimal conditions was conducted using high-performance liquid chromatography (HPLC). Furthermore, we evaluated the *in vivo* pharmacokinetics of cis- and trans-crocetin.

## 2 Materials and instruments

### 2.1 Experimental material

Gardenia yellow pigment, trans-crocetin, and cis-crocetin were made in the laboratory, and crocetin standard (content ≥ 98%) was purchased from Chengdu Pusi Bio-tech Co. Anhydrous methanol, ethyl acetate, dichloromethane, and acetic acid were purchased from Tianjin Lianlong Bohua Pharmaceutical Chemistry Co.

### 2.2 Instruments

The following instruments were used: Waters 2998 high-performance liquid chromatograph, BP210S electronic analytical balance (Sartorius), KQ-100A ultrasonic cleaner (Shanghai Ke-guide Ultrasonic Instrument Co., Ltd.), DF-1 collector-type constant-temperature heating magnetic stirrer (Changzhou Guohua Electric Co., Ltd.), SHB-IV recirculating water multi-purpose vacuum pump (Zhengzhou Great Wall Science, Technology & Industry Trade Co., Ltd.), LHH-250GSP drug stability test chamber (Shanghai Blue Leopard Experimental Equipment Co., Ltd.), AVANCE NEO 600 nuclear magnetic resonance detector, MaXis 4G ultra high-resolution mass spectrometer (Bruker), and NicoletiS10 infrared spectrometer (Thermo Fisher Scientific).

## 3 Experimental methods

### 3.1 Determination of cis-/trans-crocetin by HPLC

#### 3.1.1 Chromatographic conditions

The chromatographic column was an Elite C18 liquid chromatographic column (4.6 mm × 250 mm, 5 μm) with acetonitrile (60%): 0.1% formic acid aqueous solution (40%) as the mobile phase at a flow rate of 1.0 mL/min, the detection wavelength was 423 nm, the column temperature was 30°C, the injection volume was 10 μL, and the analysis time was 15 min.

#### 3.1.2 Solution configuration

Preparation of the control solution: The appropriate amount of cis-crocetin control was weighed in a brown volumetric flask and a small amount of DMF was added to dissolve it fully, and then methanol solution was added so that DMF: methanol = 1:20 while shaking well; that is, 10.00 mg/mL cis-crocetin control solution was taken as standby. The cis-crocetin (≥95% by HPLC) prepared in this experiment was weighed and 2.00 mg/mL of the cis-crocetin control stock solution was configured according to the above method.

Preparation of test solution: The experimentally obtained trans-crocetin was precisely weighed in a brown volumetric flask; first, a small amount of DMF was added to dissolve it fully, and then methanol solution was added so that DMF: methanol = 1:20 while shaking well; that is, 10.00 mg/mL trans-crocetin test solution was prepared the same way, and 2.00 mg/mL cis-crocetin test solution was taken as standby.

#### 3.1.3 Examination of linear relationships

The appropriate amount of trans-crocetin reference solution was accurately absorbed and diluted to obtain a series of reference solutions with varying concentrations (0.20, 0.50, 1.00, 2.00, 4.00, 6.00, 8.00, and 10.00 mg/mL). The sample was injected and determined according to the chromatographic conditions under 2.1.1. Linear regression analysis used the peak area (Y_1_) as the ordinate and the reference concentration (X_1_, mg/mL) as the abscissa.

The appropriate amount of cis-crocetin control solution was sucked up precisely and diluted to obtain a series of (0.20, 0.40, 0.60, 0.80, 1.00, 1.50, and 2.00 mg/mL) control solutions, which was injected into the sample for determination according to the chromatographic conditions under 2.1.1. Linear regression analysis was carried out with the peak area (Y_2_) as the vertical coordinate and the concentration of the control (X_2_, mg/mL) as the horizontal coordinate.

### 3.2 Preparation of high-purity trans-crocetin from gardenia yellow pigments

To obtain cis-crocetin from gardenia yellow pigment, a certain amount of the gardenia yellow pigment was weighed and mixed with a 3 mol/L NaOH solution in a material–liquid ratio of 1:6. The reaction was carried out at a constant temperature of 55°C for 60 min. After cooling to room temperature, the mixture was filtered under vacuum, and the resulting cake was dissolved in water. Further filtration was performed to remove impurities, and the filtrate was collected. The pH of the collected solution was adjusted to 2 using HCl, causing precipitation. This precipitate is crude trans-crocetin.

**FIGURE 2:**
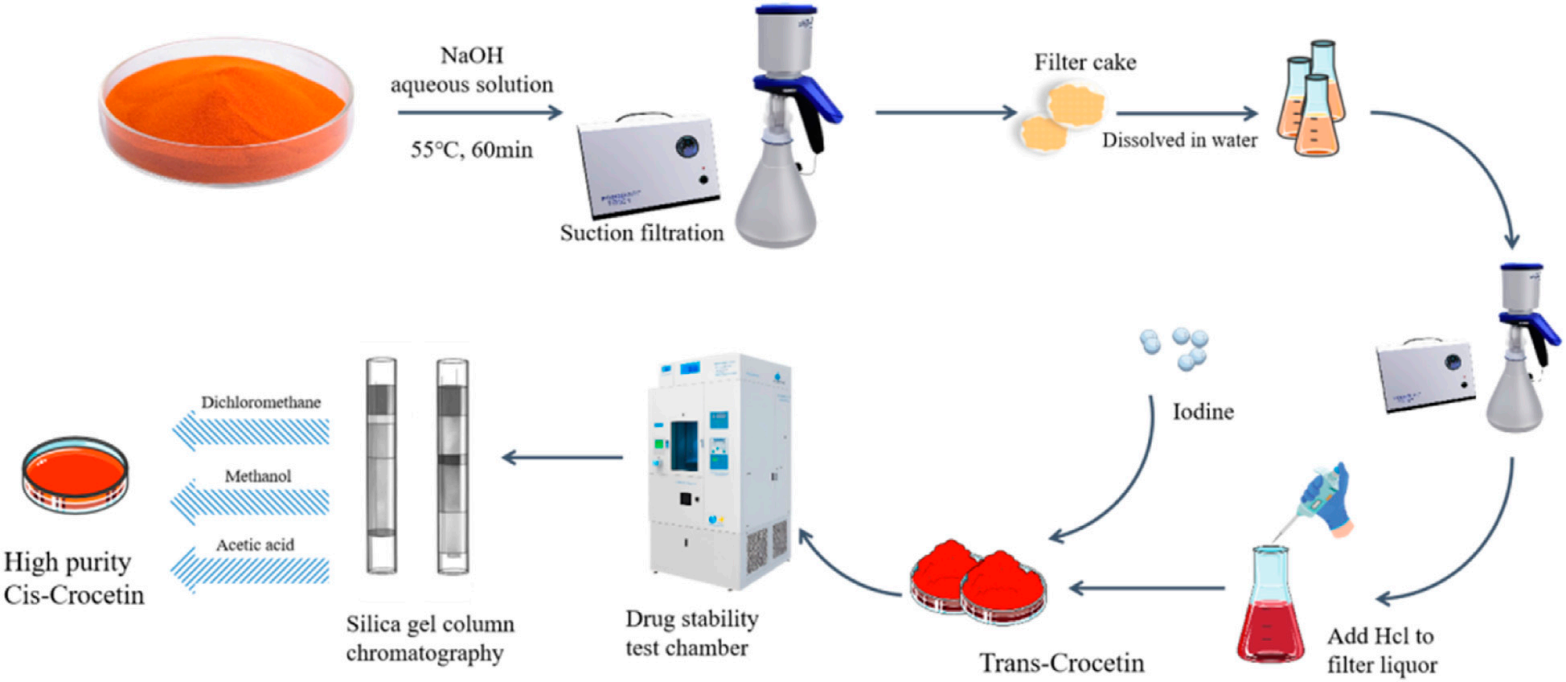
Flowchart of extracting trans- and cis-crocetin.

The crude trans-crocetin was then subjected to hydrolysis using a 10% NaOH solution for 90 min. The resulting mixture was filtered, and the trans-crocetin was collected and dissolved in water. This process is repeated twice. The solution was acidified with HCl until a red precipitate formed, and the solid was collected, yielding high-quality trans-crocetin. Subsequently, the high-purity trans-crocetin underwent iodine induction to prepare cis-crocetin (refer to Figure 2 for the process flowchart).

### 3.3 Preparation of cis-crocetin by iodine-induced transformation

To prepare the trans-crocetin–DMF solution, a specific amount of trans-crocetin was added to a DMF solution and ultrasonically dissolved, resulting in a 10.00 mg/mL concentration. Concurrently, elemental iodine was weighed and prepared in a DMF solution with the same concentration as the trans-crocetin–DMF solution. These two solutions were mixed in a 1:1 (V/V) ratio and placed in a drug stability test chamber. Illumination was provided by two 30 W light tubes positioned 30 cm away from the reaction solution. The reaction progress was monitored using HPLC, and the illumination was stopped when the reaction reached equilibrium.

**FIGURE 3:**
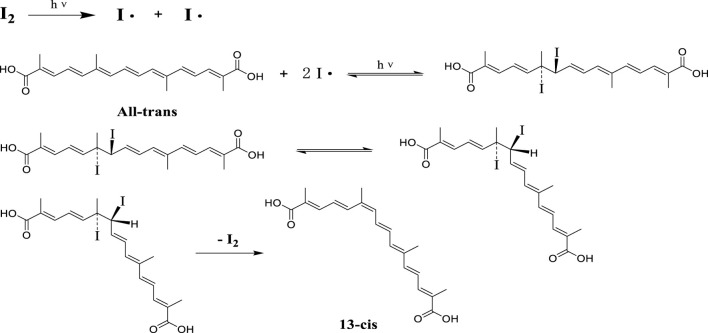
Reaction process of trans-crocetin induced by iodine.

The reaction solution was then extracted with an equal volume of ethyl acetate and washed with saturated salt water 3–4 times to remove residual DMF. The ethyl acetate layer was dried using vacuum rotary evaporation, and a small quantity of anhydrous methanol was added. After centrifugation, the supernatant was collected, and the solvent was evaporated to obtain the crude cis-crocetin. Figure 3 illustrates the conversion reaction process of trans-crocetin, as reported in the literature (Papp et al., 2001).

### 3.4 Purification of cis-crocetin

High-purity cis-crocetin was separated and prepared by silica gel column chromatography. The silica gel of 200–300 mesh was taken and mixed with dichloromethane–methanol (40:1). The mixture was placed in an ultrasonic oscillator and oscillated until there was no bubble. After pouring into the chromatography column, the column was loaded. The crude cis-crocetin obtained by the above two methods was dissolved in a small amount of methanol, and a certain amount of silica gel powder was added. After rotary evaporation and drying, the sample was loaded on a silica gel column. Using dichloromethane–methanol–glacial acetic acid (35:1:0.01) as the eluent, the thin layer chromatography was used to track and check, and the red eluent from the last layer was collected. HPLC detected the content of cis-crocetin.

### 3.5 Characterization of cis-crocetin

The molecular weight of the sample was determined by mass spectrometry, the functional groups of the sample were analyzed by infrared spectroscopy, and the molecular structure of the sample was further characterized by nuclear magnetic resonance hydrogen spectroscopy. At the same time, the content of cis-crocetin in the sample was determined by HPLC, and the sample was determined according to the chromatographic conditions under 2.1.1. The peak area and the content were calculated. The trans- and cis-crocetin were scanned by the DAD detector of high-performance liquid chromatography, and their ultraviolet absorption spectra were recorded.

### 3.6 Screening of iodine-induced transformation conditions

#### 3.6.1 Evaluation indicators of the iodine-induced transformation

The effects of the reaction solvent, iodine concentration, and trans-crocetin concentration on the process were studied by iodine-induced conversion of trans-crocetin to cis-crocetin. The maximum conversion rate, conversion rate, and isomerization rate of trans-crocetin to cis-crocetin were used as evaluation indexes to preliminarily screen out the best reaction conditions. The calculation formulas are as follows:

$$V=\frac{\Delta Z}{t},$$ (1)

$$\mathrm{CR}=\frac{\Delta Z}{\Delta E}\times \text{100}\%,$$ (2)

$$\mathrm{Is}=A_{Z}/\sum A.$$ (3)

where *V* is the conversion rate, ΔZ is the variation of cis-crocetin, ΔE is the variation of trans-crocetin, *CR* is the conversion rate, *Is* is the isomerization rate, and A is the total content of crocetin in the calculated system.

#### 3.6.2 Screening of reaction solvents

Excessive trans-crocetin was added to methanol, ethanol, ethyl acetate, and DMF solutions, and ultrasonicated for 15 min. After filtration through the filter membrane, a clear, saturated trans-crocetin solution was obtained. HPLC preliminarily determined the solubility of the solution, and the solvent with the best solubility was selected. Then, 5.0 mg of iodine was dissolved in 10 mL of the selected solvent, and 0.5 mg/mL iodine solution was prepared.

The trans-crocetin and iodine solution were mixed in the ratio 1:1 (V/V), and eight groups of experiments were carried out in parallel. The reaction solution was placed in the drug stability test box in turn. The light power was set to 30 W, and the distance from the light source was 30 cm. The light was illuminated under visible light for 0, 5, 10, 20, 30, 50, 70, and 90 min, and then 200 μL of the sample was added to 4.0 mL of anhydrous methanol. The sample was diluted and determined according to the chromatographic conditions of 2.1.1. The peak area was recorded, and the contents of cis- and trans-crocetin were calculated to determine the changes of trans- and cis-crocetin during the iodine-induced transformation.

#### 3.6.3 Screening of iodine concentration

A series of iodine–DMF solutions with concentrations (0.5, 1.0, 2.5, 5.0, and 8.0 mg/mL) were prepared. The trans-crocetin–DMF solution was mixed with different concentrations of iodine–DMF solution at a ratio of 1:1 (V/V) and placed in the drug stability test chamber. The light power was set at 30 W, and the distance from the light source was 30 cm. The light was illuminated under visible light for 0, 5, 10, 20, 30, 50, 70, and 90 min, respectively. Then, 200 μL of the sample was diluted with 4.0 mL of anhydrous methanol, and the sample was injected according to the chromatographic conditions of 2.1.1. The peak area was recorded, and the content of cis- and trans-crocetin was calculated.

#### 3.6.4 Screening of drug concentrations

A series of trans-crocetin–DMF solutions with varying concentrations (0.5, 1.0, 2.5, 5.0, and 10.0 mg/mL) were prepared. An equal 2.5 mg/mL iodine–DMF solution was added to the sample solutions with different concentrations and placed in the drug stability test box. The light power was set at 30 W, and the distance from the light source was 30 cm. The samples were illuminated under visible light for 0, 5, 10, 20, 30, 50, 70, and 90 min. Then, 200 μL of the sample was diluted with 4.0 mL of anhydrous methanol, and the sample was injected according to the chromatographic conditions of 2.1.1. The peak area was recorded, and the content of cis- and trans-crocetin was calculated.

### 3.7 Pharmacokinetic study of trans- and cis-crocetin

As the differences in conformation may bring about differences in their degree of absorption and potency in organisms, pharmacokinetic studies were designed to further investigate the differences in the metabolism of cis- and trans-crocetin *in vivo*.

#### 3.7.1 Experimental objects

The experimental animals were healthy SPF male Wistar rats 200 ± 20 g [No. SCXK (military), 2017–0023; use license no. SYXK (military) 2017–0047]. The animals were acclimatized in a rearing room with a temperature of 25°C and a relative humidity of 50% for 3 days before carrying out the experiments, with a day–night cycle of 12 h. All experimental protocols were reviewed and approved by the Animal Experimentation Committee of the Ninety-fourth Hospital of the United Logistics Force.

#### 3.7.2 Chromatographic conditions

The chromatographic column was a Waters Symmetry C18 liquid chromatographic column (4.6 mm × 150 mm, 5 μm), the gradient elution was performed with acetonitrile (A)–0.6% formic acid aqueous solution (B) as the mobile phase, and the elution procedure was as follows: 0–12 min, 10% → 70% A; 12–16 min, 70% → 85% A; 16–25 min, 85% A; 25–26 min, 85%→100% A; 26–29 min, 100% A; 29–32 min, 100%→10% A; and 32–35 min, 10% A. The flow rate was 1.0 mL/min, the detection wavelength was 423 nm, the column temperature was 37.0°C, the injection volume was 20 μL, and the analysis time was 35 min.

#### 3.7.3 Preparation of solutions

##### 3.7.3.1 Preparation of control solution

An amount of 1.5 mg of trans-crocetin control was weighed in a 25-mL volumetric flask and shaken with methanol to obtain 60.00 μg/mL of trans-crocetin control solution, which was diluted to a series of concentrations (0.10, 0.20, 0.50, 1.00, 2.00, 5.00, 10.00, 20.00, 40.00, and 60.00 μg/mL) of trans-crocetin working solution; cis-crocetin 10.00 mg was weighed precisely and diluted with methanol in a 100-mL brown volumetric flask and shaken well to obtain 100.00 μg/mL cis-crocetin control solution, respectively, which was diluted to a series of concentrations (0.10, 0.20, 0.50, 1.00, 2.00, 5.00, 10.00, 20.00, 40.00, 60.00, 80.00, and 100.00 μg/mL) of cis-crocetin working solution.

##### 3.7.3.2 Preparation of crocin-1 internal standard solution

The crocin-1 reference substance was accurately weighed in a brown volumetric flask, and the volume was fixed with methanol to obtain 10.00 μg/mL of crocin-1 internal standard solution.

#### 3.7.4 Methodological study on the determination of trans- and cis-crocetin in plasma by HPLC

##### 3.7.4.1 Specificity

A total of 80 μL of rat blank plasma was pipetted, 240 μL of methanol solution was added, with vortex mixing for 3 min, and centrifugation was performed at 4°C for 15 min at a low temperature at the speed of 12,000 r/min. The supernatant after centrifugation was aspirated in an EP tube. The solvent was evaporated in a vacuum centrifugal concentrator, and then reconstituted with 80 μL of methanol, ultrasound was performed for 5 min, vortex mixing was performed for 3 min, and then centrifugation was performed at 4°C for 10 min at a low temperature. Then centrifugation was performed at a low temperature of 4°C for 10 min at a rotational speed of 12,000 r/min, the sample was injected according to the chromatographic conditions under 2.7.2, and the chromatogram of the blank plasma was recorded.

Then 80 μL of rat blank plasma was aspirated, 240 μL of methanol was added, and then 30 μL of internal standard solution was added, and process was carried out according to the same method as above. Then the sample was injected for detection to obtain the chromatogram of blank plasma + internal standard. Approximately 80 μL of rat blank plasma was aspirated, 240 μL of methanol was added, and then 30 μL of internal standard solution and 80 μL of trans-crocetin and cis-crocetin solution were added, and processed in the same way as described above. The chromatogram was obtained after injection and detection of the blank plasma + control chromatogram.

In total, 80 μL of plasma was pipetted from rats after the administration of trans-crocetin for 1 h by gavage, 30 μL of the internal standard solution was added, and then it was processed according to the same method as described above. Then the sample was injected for determination and the chromatogram of plasma was recorded after the administration of trans-crocetin. Similarly, the plasma was pipetted from rats after the administration of cis-crocetin for 1 h by gavage and then it was processed according to the same method. Then the chromatogram of the plasma was obtained after the administration of cis-crocetin. The above chromatograms were compared to analyze whether the endogenous substances in the plasma interfered with the determination of trans- and cis-crocetin and the internal standard.

##### 3.7.4.2 Linearity and lower limit of quantification

Approximately 80 μL of blank plasma was added into the EP tube, and 160 μL of methanol solution, 30 L of internal standard solution, and 80 μL of each concentration of trans-crocetin working solution were added, respectively. Then the plasma standard curve samples were processed according to the method of the blank plasma to obtain the different concentrations (0.10, 0.20, 0.50, 1.00, 2.00, 5.00, 10.00, 20.00, 40.00, and 60.00 μg/mL) of plasma standard curve samples, according to the chromatographic conditions under 2.7.2. The concentration was taken as the horizontal coordinate (Xt), and the trans-crocetin and crocin-1 peak area ratio was taken as the vertical coordinate (Yt) for linear regression.

The plasma standard curve samples of cis-crocetin at each concentration (0.10, 0.20, 0.50, 1.00, 2.00, 5.00, 10.00, 20.00, 40.00, 60.00, 80.00, and 100.00 μg/mL) were obtained by the same method, and then injected and detected in accordance with the chromatographic conditions under 2.7.2. The concentration was taken as the horizontal coordinates (Xc), and the ratio of the peak area of cis-crocetin to crocin-1 was taken as the vertical coordinate (Yc) for linear regression.

##### 3.7.4.3 Precision

According to the operation under 2.7.3, plasma samples of trans-crocetin and cis-crocetin were prepared in three concentrations of 5.00, 20.00, and 60.00 μg/mL of quality control samples (QC), respectively, and six samples of each concentration were injected into the samples for analysis. The samples were measured within 1 day, and the measured concentrations were calculated according to linear regression equations; the intra-day precision was calculated. The samples were measured simultaneously for 3 days in a row, and the inter-day precision was calculated; the relative standard deviation was not more than 15%.

##### 3.7.4.4 Extraction recovery

According to the operation under 2.7.3, plasma samples of trans-crocetin and cis-crocetin were prepared in three concentrations of 5.00, 20.00, and 60.00 μg/mL of quality control (QC) samples, with three copies of each concentration, and then injected into the samples for analysis; another 80 L of rat blank plasma was taken from 80 L of rat; 240 L of methanol was added, vortexed for 3 min, and centrifuged at 12,000 r/min at 4°C for 15 min; and the supernatant was aspirated into another EP tube. The plasma samples of trans-crocetin and cis-crocetin were added in the high, medium, and low concentrations, and then 30 μL of the internal standard was added, vortexed for mixing, and concentrated by centrifugation at 37°C. The concentration of crocetin was then re-fused and then centrifuged at 12,000 r/min at 4°C for 15 min, and the peak areas of the internal standard in each concentration were recorded. Centrifugation was performed for 15 min, and three portions of each concentration were injected and analyzed. Then, the extraction recoveries of trans-crocetin, cis-crocetin, and the internal standard in trans- and cis-crocetin were calculated.

#### 3.7.5 Study on plasma kinetics of trans- and cis-crocetin in rats

##### 3.7.5.1 Collection of plasma samples

The trans-crocetin and cis-crocetin were fully ground into powder, and an appropriate amount of sample was added to 0.5% CMC-Na solution for vortex mixing. According to the dose of 300 mg/kg (administration volume: 1 mL/100 g), the trans-crocetin and cis-crocetin suspensions were prepared.

Twelve healthy male Wistar rats weighing 200 ± 20 g were divided into two groups, with six rats in each group. Fasting but not water was prohibited within 12 h before administration. Before administration, 300 μL of blood was collected from the fundus venous plexus as blank plasma. The rats in the two groups were given trans-crocetin and cis-crocetin by intragastric administration at a dose of 300 mg/kg. Approximately 300 μL of blood was collected from the fundus venous plexus at 10, 15, 30, 60, 120, 240, 360, 480, 720, and 1,440 min after administration and placed in a centrifuge tube containing heparin sodium. The collected blood samples were centrifuged at 4,000 r/min for 15 min to separate the upper plasma and were stored at −80°C.

##### 3.7.5.2 Treatment of plasma samples

In total, 80 μL of rat blank plasma was pipetted, 240 μL of methanol was added, and then 30 μL of internal standard solution was added, followed by vortex mixing for 3 min and centrifugation at 4°C for 15 min at a low temperature at a speed of 12000 r/min. The supernatant after centrifugation was aspirated in EP tubes, in 37°C, vacuum centrifugal concentrator to evaporate the solvent, and then 80 μL of methanol was solubilized, followed by sonication for 5 min and vortex mixing for 3 min. The supernatant was centrifuged at 12,000 r/min at 4°C for 10 min, and then the sample was injected into the sample for detection according to the chromatographic conditions under 2.7.2.

##### 3.7.5.3 Data processing and analysis

Pharmacokinetic software DAS 2.0 was used to calculate the relevant pharmacokinetic parameters; GraphPad 8.0.1 and Origin Graph 8.0 software were used to process and analyze the data and to plot the drug–time curves of trans- and cis-crocetin; SPSS 22 statistical software was used to calculate whether the results were statistically different.

### 3.8 Study on anti-hypoxia activity of trans- and cis-crocetin

Anti-hypoxic efficacy in mice was evaluated after oral administration of cis- and trans-crocetin by the atmospheric confinement hypoxia test and sodium nitrite chemical hypoxia test.

#### 3.8.1 Experimental objects

The experimental animals were healthy SPF male BABL/c mice 20 ± 2 g [use license no. SYXK (military) 2019–0010]. The animals were acclimatized in a rearing room with a temperature of 25°C and a relative humidity of 50% for 3 days before the experiments, with a day–night cycle of 12 h. All experimental protocols were reviewed and approved by the Animal Experimentation Committee of the Ninety-fourth Hospital of the United Logistics Force.

#### 3.8.2 Experimental grouping, dosage, and method

Atmospheric confinement hypoxia test: Thirty-two SPF-grade male BALB/c mice weighing (20 ± 2) g were divided into the blank control group, positive drug group, cis-crocetin group, and trans-crocetin group, with eight mice in each group. Mice in the blank control group (BG) were gavaged with 0.5% CMC-Na solution 0.1 mL/10 g for 7 days. Mice in the positive group (AC) were gavaged with acetazolamide 250 mg/kg for 7 days. Mice in the cis-crocetin group (CG) were gavaged with 140 mg/kg of cis-crocetin for 7 days. Mice in the trans-crocetin group (TG) were gavaged with 140 mg/kg of trans-crocetin for 7 days. All the above drugs were formulated in 0.5% CMC-Na solution.

After 1 h of gavage, on the seventh day, mice in each group were placed in wide-mouth bottles according to the time of gavage, and 5 g of soda lime wrapped in gauze was placed in the wide-mouth bottles to absorb the excess carbon dioxide in the bottles. The bottle stopper was thickly coated with white Vaseline. After the bottle stopper was tightly sealed, the time of death of mice was recorded to evaluate the anti-hypoxia effect.

Sodium nitrite chemical hypoxia test: The grouping and dose administered in this experiment were the same as in the atmospheric confinement hypoxia test. One hour after the end of gavage, on the seventh day, mice were injected intraperitoneally with sodium nitrite solution (300 mg/kg) in the left lower abdomen according to the sequence of gavage time. The mice were timed after the injection, and the time of death was recorded to evaluate the anti-hypoxic efficacy.

## 4 Results and analysis

### 4.1 Examination of linear relationships

The regression equation of trans-crocetin was Y_1_ = 3077012 X_1_-42377, *r* ^2^ = 0.9999, indicating that trans-crocetin had a good linear relationship in the range of 0.20–10.00 mg/mL.

The regression equation of cis-crocetin was Y_2_ = 2854926 X_2_ + 12534, *r* ^2^ = 0.9997, indicating that cis-crocetin had a good linear relationship in the range of 0.20–10.00 mg/mL.

### 4.2 Preparation of high-purity trans-crocetin from gardenia yellow pigments

After gardenia yellow pigment alkali hydrolysis generates crocetin sodium salt, cis/trans isomerization occurs, but the content of trans conformation is higher. Trans-crocetin acid is generated after acidification with HCl after the purification of successive alkali precipitation and acid extraction. The content is ≥ 95% detected by HPLC, and 50 g of gardenia yellow pigment hydrolysis can produce 8g of high-purity trans-crocetin.

### 4.3 Preparation of cis-crocetin by iodine-induced transformation

Through iodine-induced structure transformation under light conditions, iodine becomes iodine radicals under light conditions. The radicals attack the 13-position carbon-carbon double bond of trans-crocetin. An addition reaction occurs to generate iodine substitution products, probably due to the spatial resistance between iodine and methyl, resulting in the transformation of the structurally unstable part of the structure to the cis structure, which is a reversible reaction, and then cis-crocetin is generated after iodine is removed.

**FIGURE 4:**
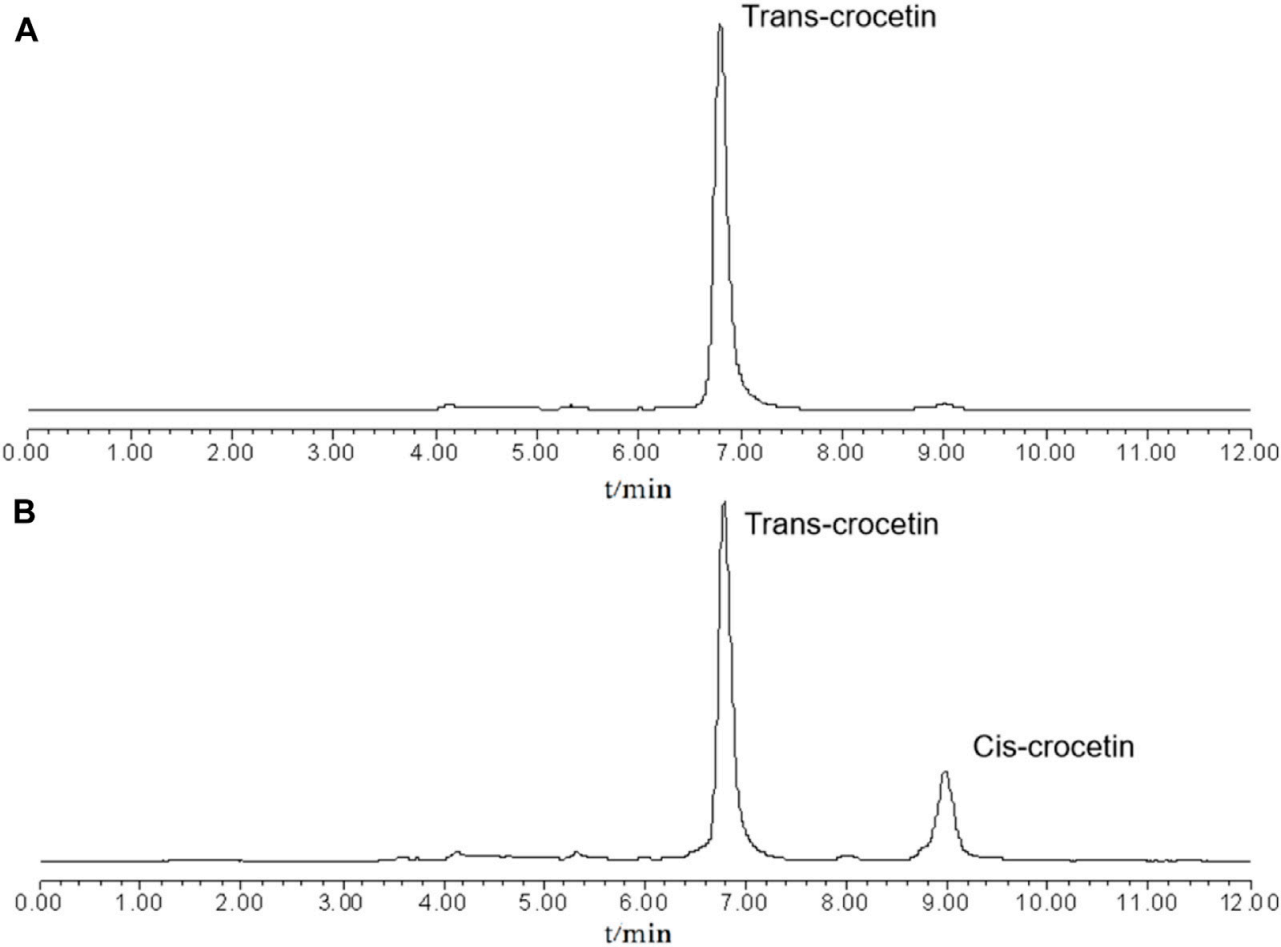
Chromatograms of the system at the beginning **(A)** and end **(B)** of the iodine-induced reaction.

Figure 4 shows the HPLC chromatograms of cis- and trans-crocetin in the system at the beginning and the end of the iodine-induced light experiments, from which it can be seen that at the beginning of the reaction stage, the system is all-trans-crocetin, and at the end of the system, cis-crocetin is around 20% of the relative peak area. Because the reaction is reversible, the amount of cis/trans-crocetin will theoretically remain stable after the reaction reaches equilibrium. However, the content of trans- and cis-crocetin will slowly decrease after reaching equilibrium due to the presence of multiple unsaturated double bonds.

### 4.4 Purification of cis-crocetin

Upon analysis of the crude cis-crocetin by HPLC, it was observed that the main impurities were a more polar sugar and some trans-crocetin. By optimizing the detection conditions, the peak positions of cis- and trans-crocetin were able to be separated, which provided insight for the subsequent separation efforts. The cis structure is characterized by two atoms or groups of atoms with the same electronegativity on the same side of the molecule, resulting in a dipole moment that is more significant than that of the trans molecules (Krohn et al., 2020). As a dipole molecule, cis-crocetin exhibits weak polarity. This observation supported the HPLC results, which showed that the peak time of trans-crocetin occurred earlier than that of cis-crocetin on the C18 column. Therefore, the difference in polarity between cis- and trans-crocetin could potentially be utilized for the separation of cis-crocetin *via* silica gel column chromatography.

When using a dichloromethane–methanol–glacial acetic acid (35:1:0.01) eluent, the large-polar sugar tends to be adsorbed on the stationary phase silica gel. Consequently, small-polar impurities are eluted first, followed by trans-crocetin, and finally, cis-crocetin. It should be noted that in cases where the crude product contains a significant amount of trans-crocetin, the solubility of trans-crocetin can lead to pronounced tailing during elution. This can result in the isolated cis-crocetin containing a certain amount of trans-crocetin. To minimize the presence of trans-crocetin, it is necessary to dry the eluate and perform another column chromatography separation. Several measures can be taken to achieve this, including reducing the material–liquid ratio during alkaline hydrolysis of gardenia yellow pigment and cooling the reaction solution to room temperature or refrigerating it. By doing so, the slightly less soluble trans-crocetin partially precipitates out, and subsequent filtration effectively reduces the content of trans-crocetin in the crude cis-crocetin product. Additionally, in the iodine-induced conversion experiments, drying the ethyl acetate layer after extraction and adding a small amount of methanol to dissolve it, followed by centrifugation, leverages the poor solubility of trans-crocetin to remove most of the trans-crocetin. This step is beneficial for the subsequent separation process.

### 4.5 Characterization of cis-crocetin

#### 4.5.1 Nuclear magnetic resonance hydrogen and carbon spectra

**FIGURE 5:**
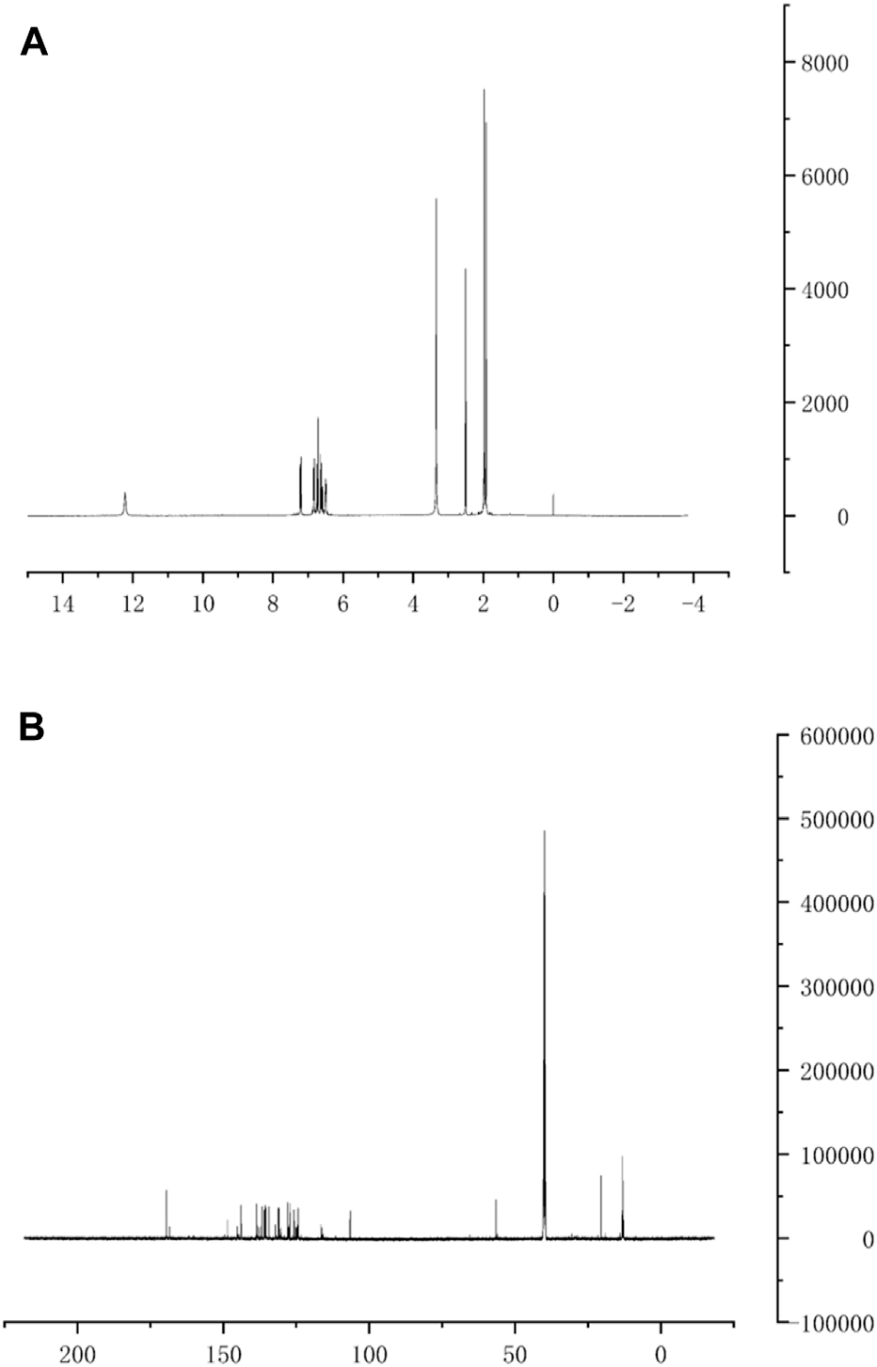
Cis-crocetin hydrogen spectrum **(A)** and carbon spectrum **(B)**.

The hydrogen spectral profile of cis-crocetin has eight groups of different types of hydrogen signals (Figure 5A): ^1^H NMR (400 MHz, DMSO)δ 12.25—12.20 (m, 2H, OH-1, OH-1′), 7.20 (d, J = 1.6 Hz, 1H, H-3), 6.84 (d, J = 3.0 Hz, 1H, H-3′), 6.73 (d, J = 15.0 Hz, 3H, H-8, H-8′, H-7), 6.61 (dd, J = 15.1, 11.3 Hz, 3H, H-5, H-5′, H-7′), 6.50 (d, J = 3.0 Hz, 2H, H-4, H-4′), 1.98 (s, 6H, 6-CH_3_, 6′-CH_3_), and 1.92 (d, J = 1.4 Hz, 6H, 2-CH_3_, 2′-CH_3_). Compared to trans-crocetin, the hydrogen spectral data were in agreement with the literature reports due to the cis isomer’s mutual coupling between hydrogen atoms in the vicinity of the isomeric position, resulting in the appearance of a variable number of multiple peaks (Yilmaz et al., 2011; Petrakis et al., 2015). Carbon spectral data of cis-crocetin (Figure 5B): ^13^C NMR (151 MHz, DMSO) δ 169.60 (d, J = 4.9 Hz, C-1, C-1′), 143.86 (t, J = 11.4 Hz, C-5, C-5′), 138.64—138.20 (m, C-3, C-3′), 135.95(s, C-6, C-6′), 135.72 (d, J = 16.2 Hz, C-7, C-7′), 131.14(s, C-8, C-8′), 125.75(s, C-2, C-2′), 124.31(s, C-4, C-4′), 20.54(s, 6-CH_3_), 13.43—13.17 (m, 2-CH_3_, 2′-CH_3_), and 12.98 (d, J = 4.8 Hz, 6′-CH_3_).

#### 4.5.2 Infrared spectroscopic analysis

**FIGURE 6:**
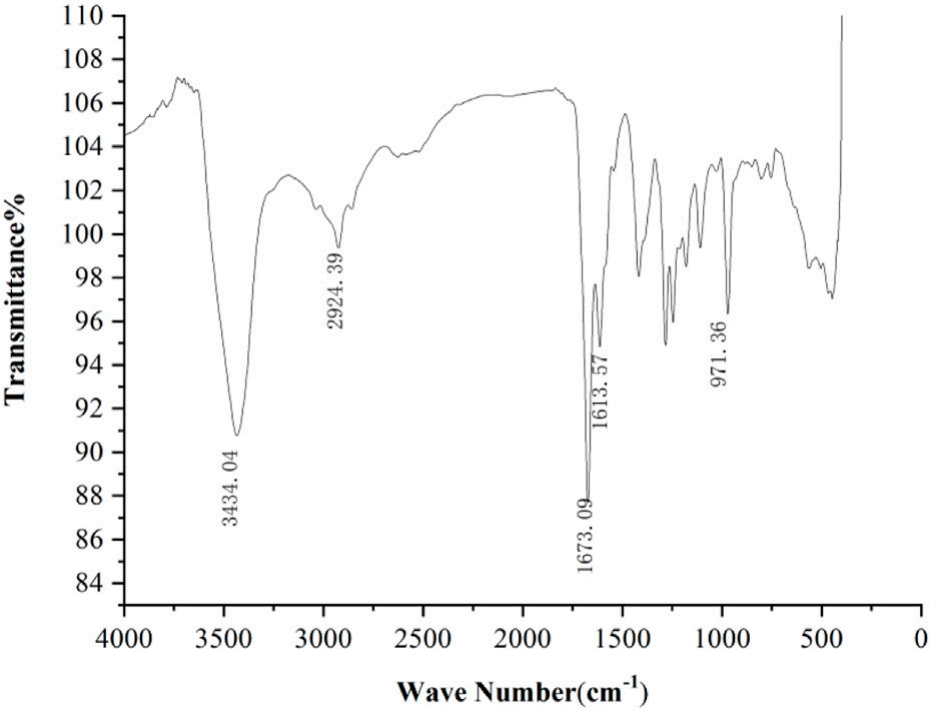
Infrared spectrum of cis-crocetin.

Figure 6 shows the FT-IR spectrum of cis-crocetin, from which it can be seen that the strong absorption peak at 3434.04 cm^-1^ is the stretching vibration of -OH on the carboxyl group; the stretching vibration of CH_3_ is near 2924.39 cm^-1^; the strong absorption peak at 1673.09 cm^-1^ corresponds to the C=O stretching vibration of carboxyl group; the strong absorption peak at 1613.57 cm^-1^ corresponds to the C=C double bond of carbon chain; the fingerprint region at 971.36 cm^-1^ corresponds to the = CH out-of-face bending vibration, which is consistent with the structure of cis-crocetin. The fingerprint region at 971.36 cm^-1^ corresponds to the = CH out-of-plane bending vibration, which is consistent with the structure of cis-crocetin (Wong et al., 2020; Saravani et al., 2023). The samples contained functional groups similar to trans-crocetin, but the peak position was red-shifted.

#### 4.5.3 Mass spectrometry

The molecular weight and molecular structure of crocetin were characterized by negative electron mode (ESI) and ion spray voltage (IS): 4000 V. Table 1 The [M-H]-peak of cis-crocetin can be found in the mass spectrum. Similar to the mass spectrum of trans-crocetin, the intensity of the ion peak [M-1–138] is the strongest, and the position of its fracture is the position of cis-trans isomerization in crocetin, that is, the peak produced by the fracture of the 13-position carbon–carbon double bond.

**TABLE 1:** Negative ion mode mass spectrometry data.

Crocetin	Main ion fragment peaks (m/z)				
M 328	[M-H]-327	[M-45]-283	[M-1–62]-265	[M-1–72]-255	[M-1–138]-189

#### 4.5.4 Determination of content by HPLC and analysis of UV-absorption spectra

**FIGURE 7:**
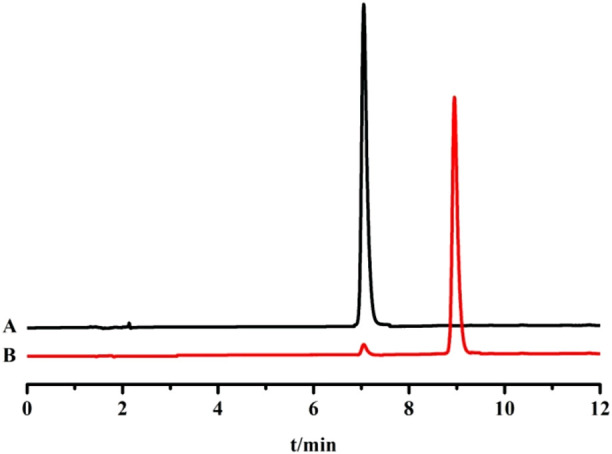
HPLC chromatograms of trans-crocetin reference substance **(A)** and cis-crocetin sample **(B)**.

The prepared samples were injected and analyzed under the chromatographic conditions of 2.1.1. The chromatograms are shown in Figure 7. The separation of trans- and cis-crocetin is good. The content of cis-crocetin can be preliminarily estimated by the normalization method to reach more than 95%. Currently, there is no product of cis-crocetin reference substance on the market. Therefore, the self-made cis-crocetin was used as the reference substance in the follow-up experiment to carry out the subsequent iodine-induced transformation experiment.

**FIGURE 8:**
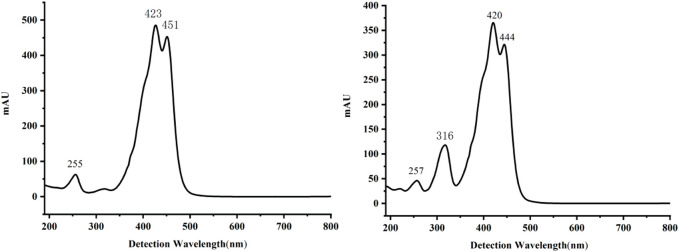
UV absorption spectra of trans (left) and cis (right) crocetin.

Figure 8 shows the UV absorption of trans- and cis-crocetin in the range of 190–800 nm. As shown in Figure 8, the trans and cis structures of crocetin have two firm absorption peaks in the 400–450 nm range and weak UV absorption at about 255 nm. However, compared with trans-crocetin, cis-crocetin has a prominent ultraviolet absorption peak at 316 nm, that is, “cis-peak.” The reason may be that the cis leads to the change of its conjugated structure, which causes it to produce other conjugated structures and thus has ultraviolet absorption at 310 nm. Therefore, the peak area of trans-crocetin is slightly larger than that of cis-crocetin at the same concentration, which is also consistent with the measurement results.

### 4.6 Screening of iodine-induced transformation conditions

#### 4.6.1 Screening of reaction solutions

**FIGURE 9:**
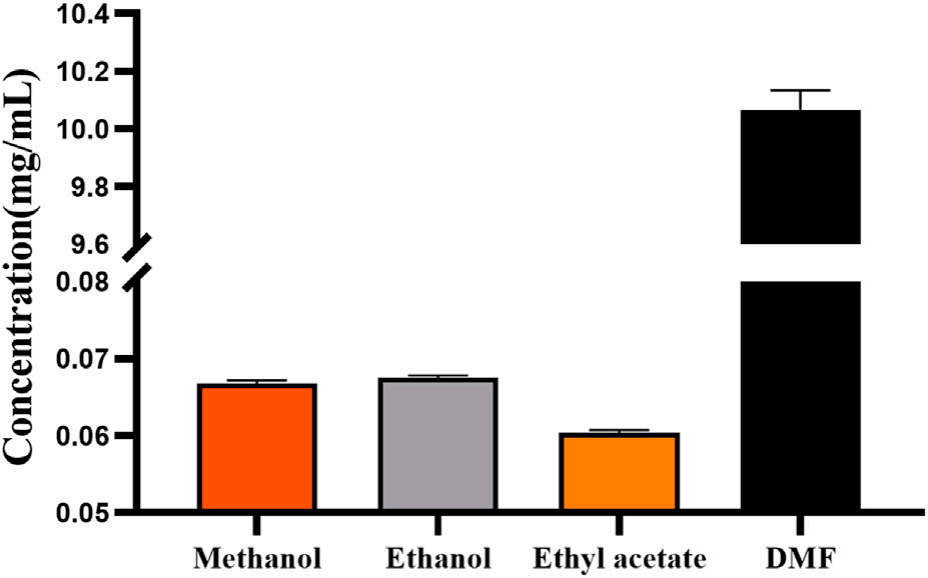
Solubility of trans-crocetin in different solvents.

To meet the requirements for the preparation of cis-crocetin, the solvent chosen should have high solubility and, at the same time, be able to effectively meet the iodine-induced reaction and maintain the stability of trans- and cis-crocetin during the reaction to minimize the loss of the reaction. From Figure 9, it can be seen that trans-crocetin has the best solubility in DMF solution, so DMF as a solvent can meet the experiment’s needs.

**FIGURE 10:**
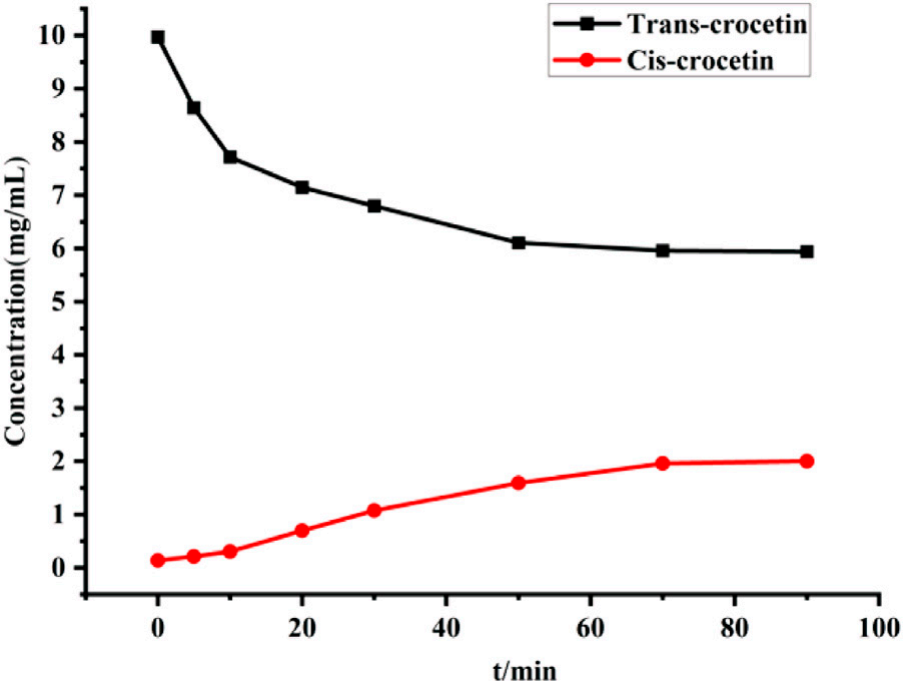
Changes in trans- and cis-crocetin concentrations during iodine-induced transformations.

The peak areas of trans- and cis-crocetin were recorded at each time point, and their concentrations were calculated after substituting into the standard curve, as shown in Figure 10. With the extension of the light time, the concentration of trans-crocetin in the system first declined rapidly, and then the concentration tended to be stabilized at the time of light time of 70 min; on contrary, the concentration of cis-crocetin first increased rapidly with the extension of the light time, and then it tended to be stabilized at 70 min. From the results, it can be seen that the trend of cis-crocetin is opposite to that of trans-crocetin, indicating that the trans structure in the system was induced by iodine to generate the cis structure gradually. The concentration can be stabilized after a particular time of illumination, which indicates that the experimental requirements can be met when DMF is used as the solvent.

#### 4.6.2 Screening of iodine concentration

Crocetin has a polyunsaturated structure that makes it susceptible to decomposition under iodine-induction and light-exposure conditions. This can result in a decreased conversion rate of cis-crocetin, leading to significant losses of raw materials and products. Therefore, it is necessary to screen the iodine concentration during the conversion process to minimize these losses. It should be noted that the duration of light exposure also affects the reaction process. A short time of rapid conversion may not be suitable for accurately monitoring the reaction process, whereas prolonged exposure may increase production costs and reduce efficiency due to poor stability. Therefore, it is important to screen the iodine concentration and adjust the light exposure time accordingly to minimize losses of raw materials and products. By doing so, the reaction process can be more efficiently controlled.

**FIGURE 11:**
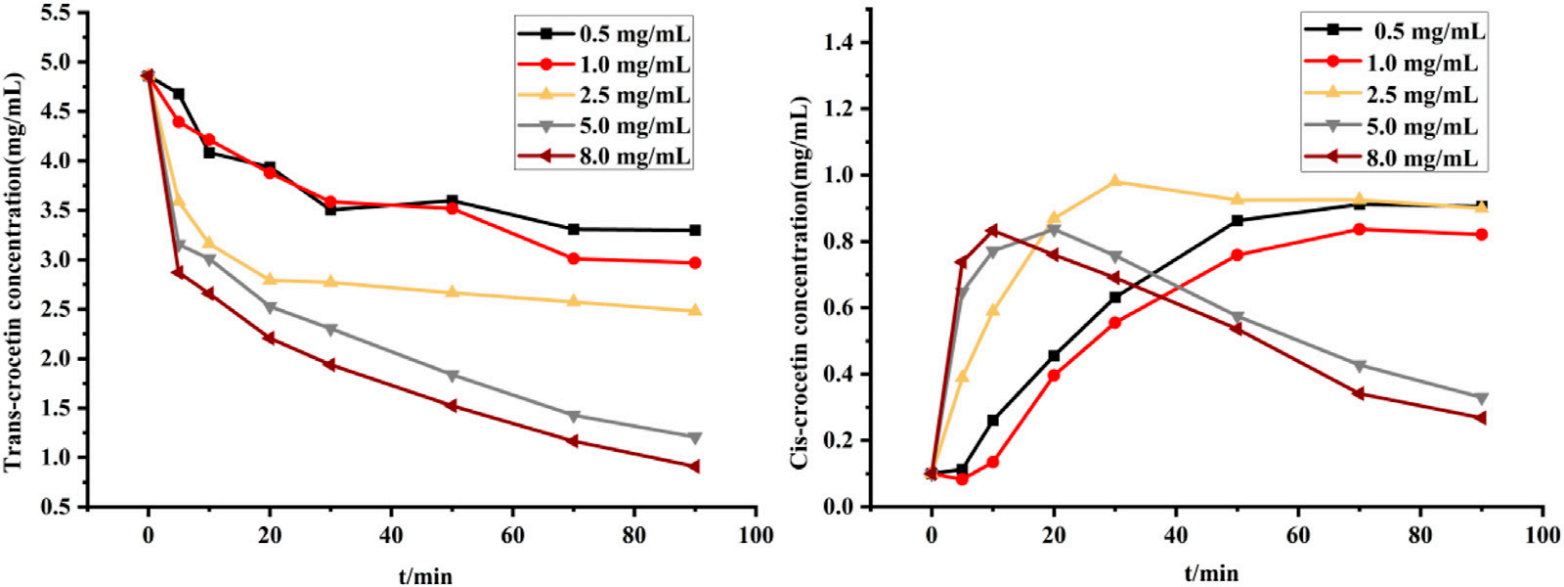
Changes in trans- and cis-crocetin at different iodine concentrations.

Figure 11 illustrates the changes in trans- and cis-crocetin concentrations when different concentrations of iodine solutions were used to induce the reaction. The results indicate that as the iodine concentration increases, the concentration of trans-crocetin decreases at a faster rate. This suggests that higher iodine concentrations result in faster reaction rates, but it also implies that excessively high iodine concentrations can lead to the decomposition of trans-crocetin and a decrease in its structural stability over time. Conversely, when the iodine concentration is low, the cis-crocetin content in the system slowly increases with an extended illumination time.

However, when the iodine concentration is high, the cis-crocetin concentration reaches its maximum quickly before rapidly decreasing. By examining the changes in trans- and cis-crocetin, we conclude that an iodine concentration of less than 2.5 mg/mL results in relatively stable trans- and cis-crocetin structures but requires a more extended period to reach equilibrium conversion. By contrast, higher iodine concentrations lead to a faster conversion of cis-crocetin but are characterized by the decreased stability of both trans- and cis-crocetin. We therefore chose an iodine solution with an iodine concentration of 2.5 mg/mL for the reaction as it resulted in a shorter time to reach equilibrium conversion, with minimal changes in both trans- and cis-crocetin contents over time.

#### 4.6.3 Screening of drug concentration

**FIGURE 12:**
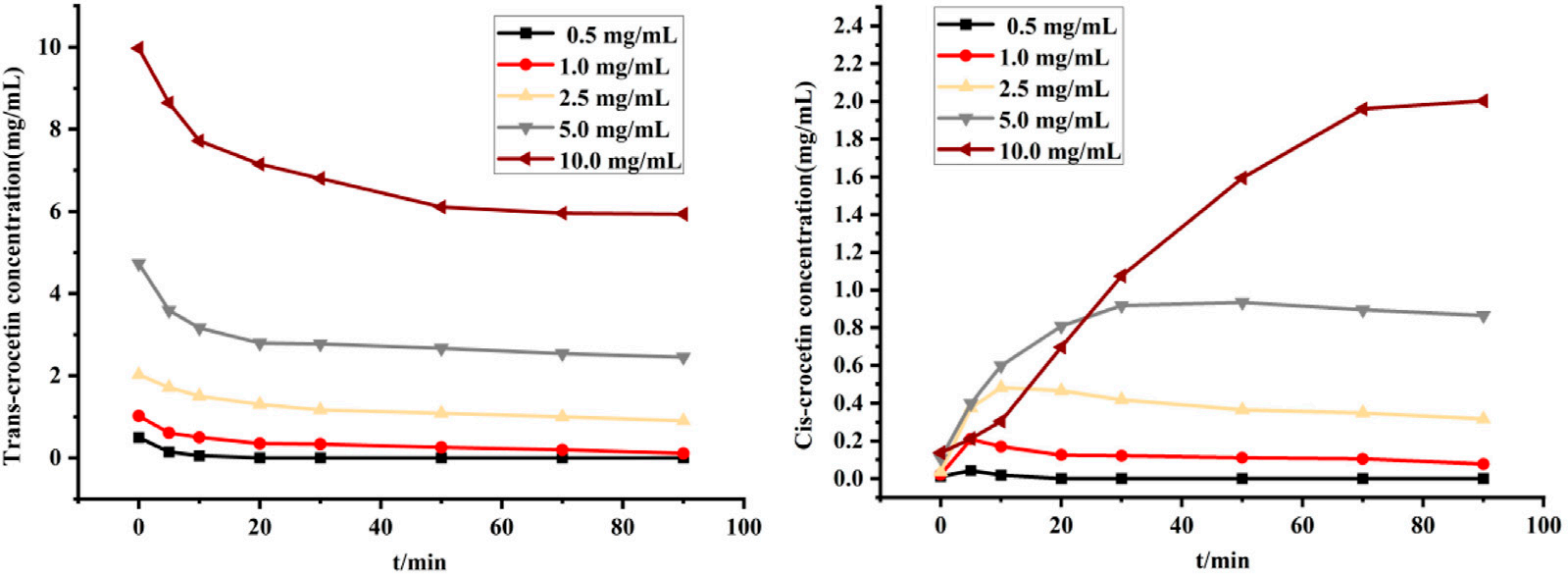
Variation of trans- and cis-crocetin at different drug concentrations.

Figure 12 shows the variation of trans- and cis-crocetin concentrations when different concentrations were used for the reaction. As can be seen from the figure, when the drug concentration was less than 2.5 mg/mL, both trans- and cis-crocetin contents decreased rapidly, and trans-crocetin was converted to cis-crocetin in a very short time before it was utterly decomposed; meanwhile, the cis content briefly increased and then decreased to zero, which was probably because of the addition of an equal volume of 2.5 mg/mL iodine solution when the drug concentration was low. The system contained a relatively large amount of iodine, which led to an unstable decomposition of both the cis and the trans structures. When the drug concentration was more significant than or equal to 2.5 mg/mL, a longer time was required to reach equilibrium for the trans and cis transformations as the concentration increased, and the greater the drug concentration, the greater the concentration of cis-crocetin after reaching equilibrium.

The conversion rate, Conversion velocity, and isomerization rate were used as evaluation indexes to further evaluate the efficiency of the conversion of trans-crocetin to cis-crocetin at different drug concentrations. As shown in Table 2, the isomerization rate in the system did not differ much at different drug concentrations when the cis-content was at its maximum, which was related to the reversibility of the reaction and the stability of cis-/trans-crocetin. When the drug concentration was 2.5 mg/mL, the maximum conversion rate of cis-crocetin reached 86.13%, which indicated that the stability of trans- and cis-crocetin was better during the conversion process. Most of the trans-crocetin was converted to the cis configuration. The maximum conversion rate was 44.60 μg/min. Combining the screening results of all the conditions, the solvents of the reaction were chosen to be DMF, and the concentrations of both trans-crocetin and the iodine solutions were chosen to be DMF. Crocetin and iodine solutions were both at a concentration of 2.5 mg/mL, and the light time could be adjusted appropriately with the content of trans-crocetin.

**TABLE 2:** Conversion rate, Conversion velocity and isomerization of cis-crocetin at different drug concentrations.

Drug concentration (mg/mL)	CR (%)	V (μg/min)	Is (%)
0.5	9.09	6.42	23.17
1	44.86	37.41	25.58
2.5	86.13	44.60	24.33
5	54.76	20.01	27.64
10	46.22	20.72	25.22

Note: CR: conversion rate; V: conversion velocity; IS: isomerization rate.

### 4.7 Pharmacokinetic study results of trans- and cis-crocetin

#### 4.7.1 HPLC detection methodology inspection results

##### 4.7.1.1 Specificity

**FIGURE 13:**
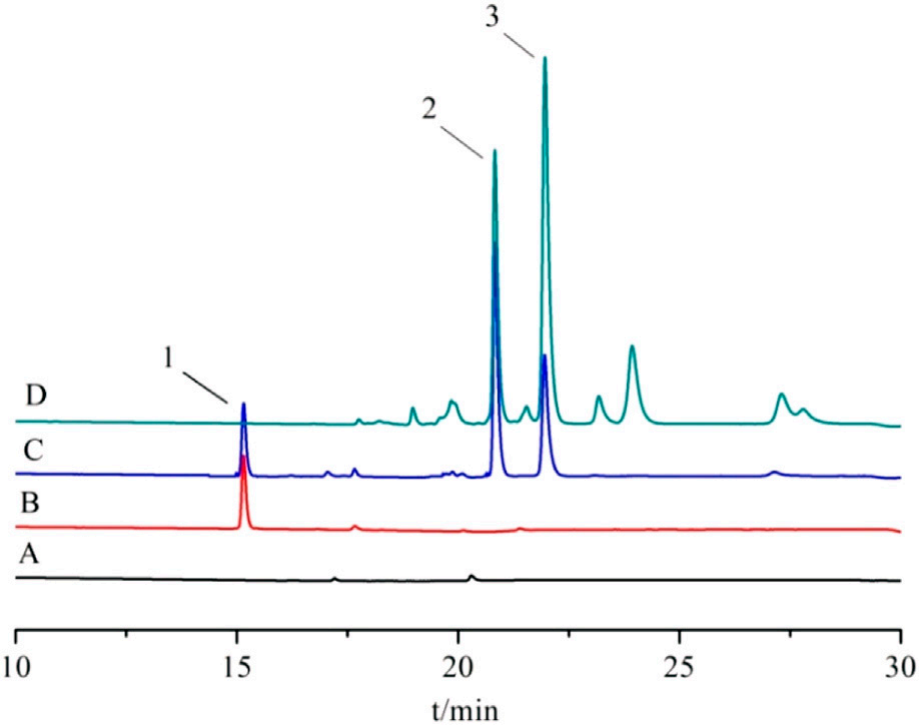
High-performance liquid chromatogram. Note: **(A)** blank plasma; **(B)** blank plasma + internal standard; **(C)** blank plasma + reference substance; **(D)** plasma samples; 1. Crocin-1 internal standard; 2. trans-crocetin; 3. cis-crocetin.

Appropriate amounts of the above blank plasma, control solution, and plasma sample solution were taken. They were detected by high-performance liquid chromatography, and the chromatograms were recorded. The results are shown in Figure 13. There was no interference of the heterogeneous peaks in the blank plasma, and under this chromatographic condition, the separation of trans- and cis-crocetin in the plasma was good and with good peak shape.

##### 4.7.1.2 Linearity and lower limit of quantification

The regression equation for trans-crocetin was Yt = 0.8508Xt+0.5075, r2 = 0.9986, indicating that trans-crocetin had a good linear relationship in the range of 0.10–100.00 μg/mL, and that for cis-crocetin was Yc = 0.5091Xc-0.1691, r2 = 0.9983, indicating that cis-crocetin had a good linear relationship in the range of 0.10–60.00 μg/mL. For trans-crocetin, the lower limit of quantification was 0.10 μg/mL, the RSD was 5.91%, and the RE was 8.45%, and for cis-crocetin, the lower limit of quantification was 0.10 μg/mL, the RSD was 7.54%, and the RE was 12.11%.

##### 4.7.1.3 Precision and recovery rate

The intra- and inter-day RSD of trans-crocetin were in the range of 1.41%–6.54%, and the RE were in the range of 3.41%–7.36%; the intra- and inter-day RSD of cis-crocetin were in the range of 1.43%–7.17%, and the RE were in the range of 2.97%–7.39%, which showed that the method had good precision.

The extraction recoveries of trans- and cis-crocetin at high, medium, and low concentrations were in the range of 88.28–94.00%, in which the extraction recoveries of the internal standard were 89.08–96.66%. The results showed that the extraction recoveries of trans- and cis-crocetin in plasma were good, and the recoveries of the added internal standard were good, which met the requirements for the analysis of biological samples.

#### 4.7.2 Plasma kinetic results of trans- and cis-crocetin in rats

##### 4.7.2.1 Plasma drug concentration–time curve

**FIGURE 14:**
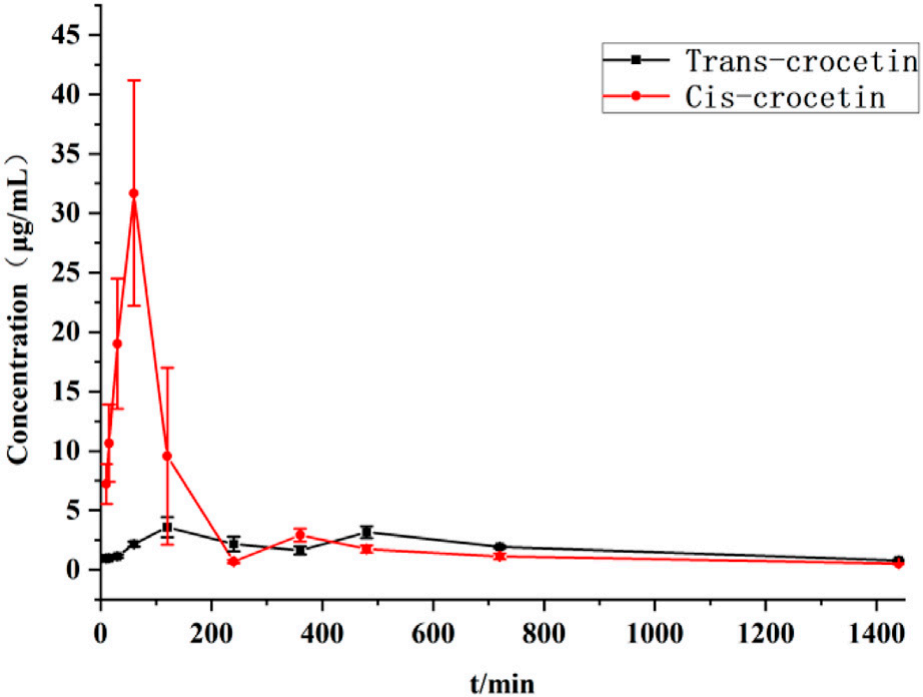
Plasma concentration–time curves of trans- and cis-crocetin in rats after intragastric administration.

The blood concentration–time curves of trans-crocetin and cis-crocetin administered by gavage to rats at the same dose are shown in Figure 14, from which it can be seen that trans-crocetin showed an obvious “bimodal” phenomenon, and although the relative cis-crocetin was also absorbed bimodally, the blood concentration had decreased significantly at the time of the second peak, which could be attributed to its conversion into trans-crocetin and its rapid metabolism in the body. Although cis-crocetin also had bimodal absorption, the blood concentration had decreased significantly at the second peak, probably because it was converted to trans-crocetin and rapidly metabolized *in vivo*. Meanwhile, the peak time of cis-crocetin was earlier than that of trans-crocetin, and the blood concentration of cis-crocetin was much larger than that of trans-crocetin.

**FIGURE 15:**
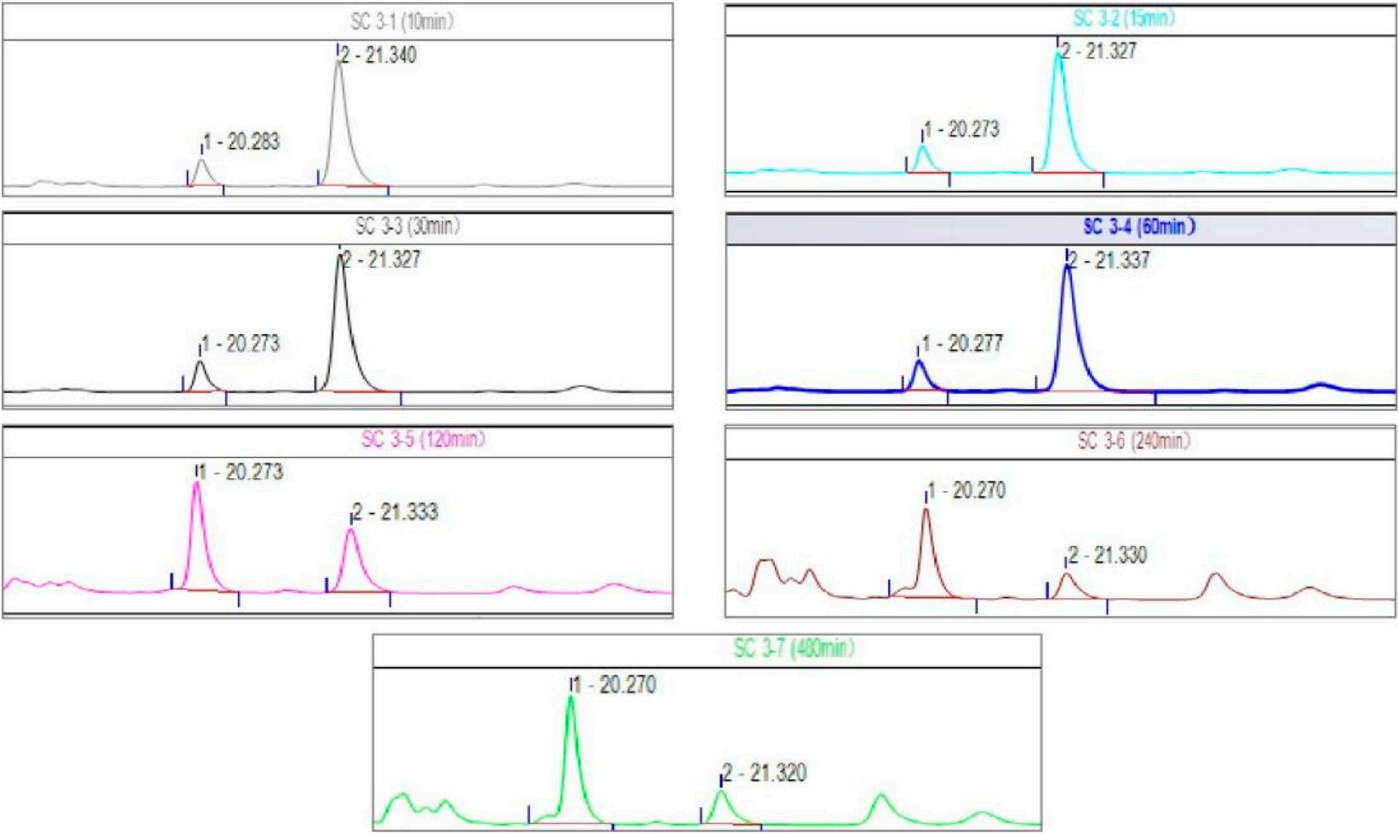
HPLC chromatograms of cis-crocetin at different times after intragastric administration.

In addition, after the administration of cis-crocetin by gavage to rats, besides the chromatographic peak of cis-crocetin found in plasma, there was another obvious chromatographic peak, as shown in Figure 15, which shows the HPLC spectra of the administration time from 10 to 480 min, and it can be seen that the area of this unknown peak showed an upward trend within a short period of time after the administration of cis-crocetin, and the trend of the change over time was in consistency with that of cis-crocetin. By comparing the peak appearance time of this unknown peak with that of trans-crocetin, it was found to be the same, and further comparing its UV absorption with that of trans-crocetin, it was confirmed that this unknown peak was trans-crocetin.

**FIGURE 16:**
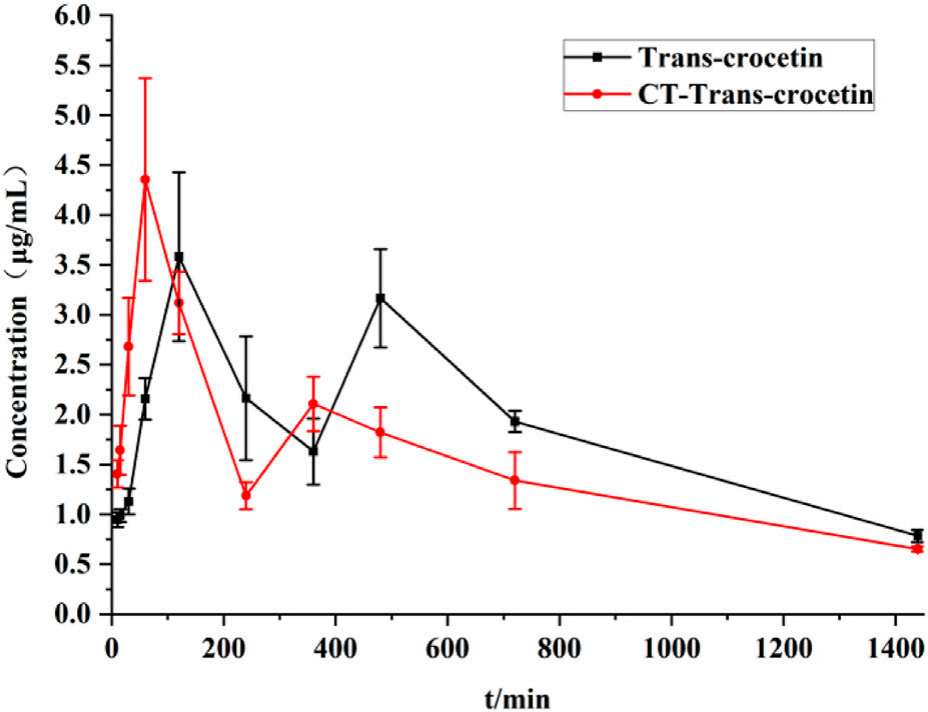
Blood concentration–time curves of trans-crocetin and CT-trans-crocetin.

After the administration of cis- and trans-crocetin, the peak area converted to trans-crocetin (CT-trans-crocetin) was recorded at each time point, substituted into the standard curve for trans-crocetin, and the blood concentration–time curve was calculated and plotted, and compared with that of the blood concentration–time curve for the direct administration of trans-crocetin, as shown in Figure 16. The CT-trans-crocetin also contained a double peak, which was similar to that of the cis- and trans-crocetin’s drug–time curve with the same trend. The concentration of the second peak would be significantly lower than that of the direct administration of trans-crocetin, but the overall peak area under the curve was closer.

##### 4.7.2.2 Pharmacokinetic parameters

The main pharmacokinetic parameters such as the area under the blood concentration–time curve (AUC), terminal clearance (CL_z_), terminal half-life (t_1/2z_), terminal apparent volume of distribution (V_z_/F), and mean retention time (MRT) were determined after gavage administration of trans- and cis-type crocetin. The results are shown in Table 3. The maximum time to peak was about 2 h for trans-crocetin and about 1 h for cis-crocetin, indicating that cis-crocetin was absorbed more rapidly than trans-crocetin, whereas the blood concentration C_max_ at the time of peak of cis-crocetin was much greater than that of trans-crocetin. However, the relative CL_z_, t_1/2z_, V_z_/F, and MRT of the cis-form would be smaller than that of the trans-form.

**TABLE 3:** Pharmacokinetic parameters of cis- and trans-crocetin in rats after intragastric administration.

Parameter	cis-crocetin	trans-crocetin	CT-trans-crocetin
AUC(0-t)/mg·L^-1^·h^-1^	68.25 ± 10.85^a,c^	45.49 ± 10.60	36.11 ± 3.31
AUC(0-∞)/mg·L^-1^·h^-1^	72.13 ± 13.70^b,c^	58.38 ± 7.34	46.29 ± 3.04
MRT (0-t)/h	4.58 ± 0.36	9.42 ± 2.21	-
MRT (0-∞)/h	6.16 ± 1.26	16.34 ± 3.08	-
t_1/2Z_/h	6.69 ± 3.07	11.36 ± 3.82	-
T_max_/h	1.17 ± 0.41	2.33 ± 0.82	-
V_Z/F_/L·kg-1	38.98 ± 12.93	82.71 ± 18.31	-
CL_Z/F_/L·h^-1^·kg^-1^	4.30 ± 0.91	5.20 ± 0.61	-
C_max_/mg·L^-1^	32.45 ± 8.48	3.80 ± 0.45	4.37 ± 1.00

Note: T_max_: drug peak time; C_max_: drug peak concentration: a indicates *p* < 0.001 compared with the trans-crocetin group, b indicates *p* < 0.05 compared with the trans-crocetin group, and c indicates AUC _(Cis+CT-Trans)_ *p* < 0.001 compared with the trans-crocetin group.

The blood concentration of CT-trans-crocetin was calculated, and its AUC was obtained by preliminary analysis using DAS 2.0 software, but the pharmacokinetic parameters related to CT-trans-crocetin including CL_z_, t_1/2z_, V_z_/F, and MRT were not analyzed as they were converted to trans-crocetin after the administration of cis-crocetin.

### 4.8 Results on anti-hypoxia activity of trans- and cis-crocetin

#### 4.8.1 Atmospheric confinement hypoxia test

**FIGURE 17:**
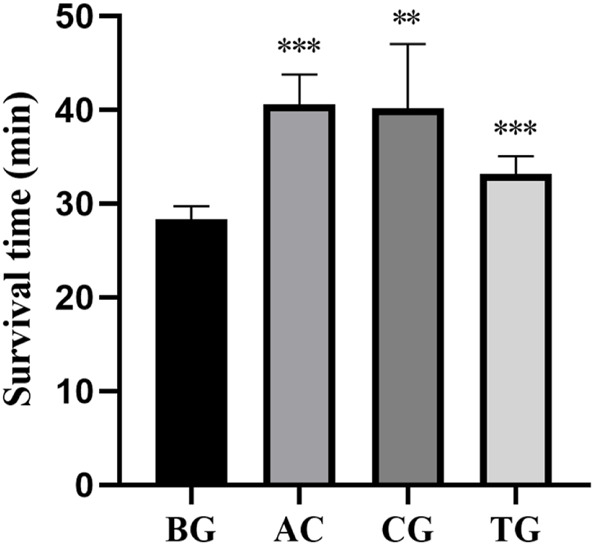
Survival time of atmospheric confinement hypoxia test. Note: *: *p* < 0.05, **: *p* < 0.01, ***: *p* < 0.001.

As shown in Table 4, acetazolamide, cis-crocetin, and trans-crocetin prolonged the hypoxic survival time in the atmospheric confinement experiment in mouse. The lengthening rates were 43.251%, 41.978%, and 17.210%, respectively. Compared with the BC group, AC and TC groups had extremely significant statistical differences. There was a statistically significant difference between the CG group and the BC group (Figure 17).

**TABLE 4:** Survival time of mice in atmospheric confinement hypoxia test ( $\bar{x}$ ± s, n = 8).

Group	Mean	SD	Lengthening rate (%)
BG	28 min 18 s	1 min 26 s	-
AC	40 min 33 s	3 min 15 s	43.25
CG	40 min 11 s	6 min 50 s	41.98
TG	33 min 11 s	1 min 54 s	17.21

Note: BG, blank control group; AC, positive group; CG, cis-crocetin group; TG, trans-crocetin group.

#### 4.8.2 Sodium nitrite chemical hypoxia test

**FIGURE 18:**
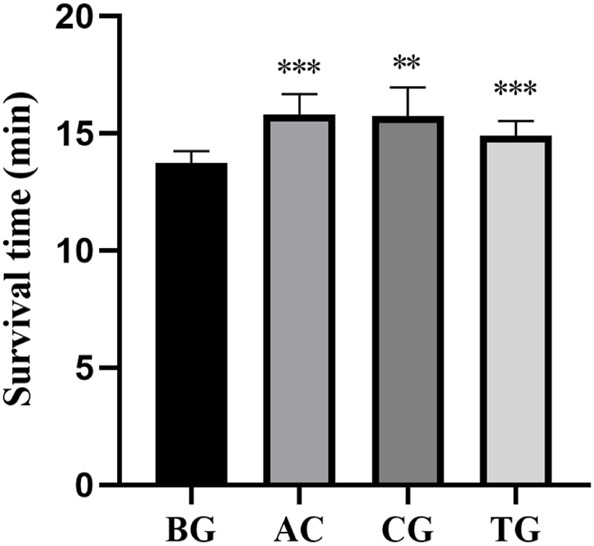
Survival time of sodium nitrite chemical hypoxia test. Note: *: *p* < 0.05, **: *p* < 0.01, ***: *p* < 0.001.

As shown in Table 5, acetazolamide, cis-crocetin, and trans-crocetin prolonged the survival time in the sodium nitrite chemical hypoxia test in mouse. The lengthening rates were 15.101%, 14.630%, and 8.469%, respectively. Compared with the BC group, AC and TC groups had extremely significant statistical differences. There was a statistically significant difference between the CG group and the BC group (Figure 18).

**TABLE 5:** Survival time of mice in sodium nitrite chemical hypoxia test ( $\bar{x}$ ± s, n = 8).

Group	Mean	SD	Lengthening rate (%)
BG	13 min 44 s	31 s	-
AC	15 min 48 s	53 s	15.10
CG	15 min 44 s	1 min 14 s	14.63
TG	14 min 53 s	39 s	8.47

Note: BG, blank control group; AC, positive group; CG, cis-crocetin group; TG, trans-crocetin group.

## 5 Conclusion

High-purity trans-crocetin was obtained by alkaline hydrolysis of gardenia yellow pigment. Then, iodine was used to induce trans-crocetin to obtain cis-crocetin, and the structure of the resulting product was identified as the target product. The optimal reaction conditions were initially screened using the maximum conversion rate of trans to cis, the conversion rate, and the isomerism of the isomers as evaluation indexes. DMF was selected as the solvent of the reaction, with trans-crocetin and iodine solution both at a concentration of 2.5 mg/mL, and the light time was appropriately increased with the content of trans-crocetin. High-purity cis-crocetin was then isolated by silica gel column chromatography. The cis-crocetin can be stabilized in the solid form at room temperature and pressure.

The pharmacokinetic experiments of trans- and cis-crocetin revealed that cis-crocetin was absorbed more readily in the body than trans-crocetin. The peak time of cis-crocetin was 1 h earlier than that of trans-crocetin, and the maximum blood concentration was 8.5 times higher than that of trans-crocetin. The areas under the concentration–time curves of cis-crocetin were greater than those of trans-crocetin, suggesting that cis-crocetin was more rapidly absorbed in the body. However, the half-life of cis-crocetin became shorter, and its blood concentration at the second peak was already reduced to 9.23% of the first.

We found that cis-crocetin and trans-crocetin prolonged the survival time of experimental animals and showed significant anti-hypoxic activity, both for atmospheric confinement hypoxia and chemical hypoxia by reducing the oxygen binding rate of hemoglobin through sodium nitrite.

Overall, the results indicate that cis-crocetin is more effectively absorbed in the body than trans-crocetin, with a faster rate of absorption. In addition, cis-crocetin showed notable anti-hypoxic activity.

## 6 Summary

Trans-crocetin has demonstrated various pharmacological effects, but its insolubility limits absorption in organisms and results in poor bioavailability. To address this solubility issue, the structure of crocetin is often modified to increase solubility. In our preliminary experiments, we found that alkali hydrolysis can be used to obtain the sodium salt of crocetin, which improves the aqueous solubility of trans-crocetin and thus enhances its bioavailability. Additionally, our research group discovered that cis-crocetin exhibits superior lipid solubility compared to its trans-configuration, making it more favorable for absorption and utilization in the body.

We observed that the content of natural cis-crocetin is limited, with the yield of cis-crocetin obtained from gardenia yellow pigment being only approximately 0.6%, compared to the yield of trans-crocetin, which can be more than 15%. Various methods can be utilized to convert the trans configuration to the cis configuration. Currently, cis and trans configuration conversion methods primarily consist of thermogenic isomerization, heat-promoted isomerization, and photo-isomerization reactions. During our stability studies of cis/trans-crocetin, we observed that heat treatment has minimal effect on the cis/trans-configuration. Conversely, the heat-promoted isomerization reaction requires the addition of a solid oxidizing catalyst during the reaction, a factor that can limit subsequent product treatments (Shi et al., 2002; Colle et al., 2016). The photoisomerization reaction typically employs iodine as a photosensitizer, which facilitates a controllable reaction process and easy product separation. However, during our attempts to convert trans- to cis-crocetin using UV light, we found that the results were contrary to our expectations, which may be due to the higher energy of the cis structure being converted to the lower energy and more stable trans conformation under UV light. Therefore, after reviewing the literature, we partially converted trans- to cis-crocetin by employing an iodine-induced method under visible light illumination conditions.

Cis-crocetin was synthesized through an iodine-induced conversion method, utilizing trans-crocetin as the starting material. Several factors, including reaction solvent, iodine concentration, and trans-crocetin concentration, were screened to optimize the reaction conditions. The evaluation criteria for selecting the preliminary reaction conditions included the conversion rate of cis-crocetin, the isomerization rate, and other relevant indicators. Subsequently, high-purity cis-crocetin was isolated through silica gel column chromatography.

The maximum absorbed concentration of cis-crocetin was reached after approximately 1 h, which was 1 h earlier than that of trans-crocetin. Additionally, the area under the curve for cis-crocetin was larger than that for trans-crocetin. It is worth noting that the half-life of cis-crocetin was shorter; however, upon administration, cis-crocetin undergoes conversion to the trans-isomer within the body, with approximately 80% of the administered cis-crocetin being converted to trans-crocetin. This suggests that cis-crocetin can be rapidly absorbed to exert its therapeutic effect. Furthermore, after partial conversion to the trans-isomer, it exhibits an increased residence time in the body, thereby prolonging the drug’s half-life to a certain extent. Subsequent experiments can consider modifying the dosage form or employing other methods to further prolong the drug’s half-life. In this study, we found that cis-crocetin possesses notable anti-hypoxic efficacy. Moreover, it is important to explore the pharmacological activity of cis-crocetin and determine whether it differs from the efficacy of trans-crocetin through additional experimental studies. Many existing studies have indicated that the efficacy of trans-crocetin is associated with its antioxidant mechanism *in vivo* (Hashemi and Hosseinzadeh, 2019).

## Data Availability

The raw data supporting the conclusion of this article will be made available by the corresponding author, LM, upon reasonable request.

## Abbreviations

^13^C NMR, nuclear magnetic resonance carbon spectrum; ^1^H NMR, nuclear magnetic resonance hydrogen spectrum; AUC, area under the blood concentration–time curve; CL/z, terminal clearance; CMC-Na, carboxymethylcellulose sodium; DMF, N,N-dimethylformamide; ESI, electron mode; FT-IR, Fourier-transform infrared spectroscopy; HPLC, high-performance liquid chromatography; IS, ion spray; MRT, mean retention time; QC, quality control; t_1/2z_, terminal half-life; UV, ultraviolet; V_z_/F, terminal apparent volume of distribution.
